# Re: Indigenous people’s experiences of primary health care in Canada: a qualitative systematic review

**DOI:** 10.24095/hpcdp.44.7/8.06

**Published:** 2024-07

**Authors:** Chandrakant P. Shah

**Affiliations:** 1 Professor Emeritus, Dalla Lana School of Public Health, University of Toronto, Toronto, Ontario, Canada; 2 Honorary Consulting Physician, Anishnawbe Health Toronto, Toronto, Ontario, Canada

Dear Editor, 

I read the article by G. Barbo and S. Alam titled “Indigenous people’s experiences of primary health care in Canada: a qualitative systematic review,” which was published in the April issue of your journal.^1^ As someone who has been involved in providing primary care to the Indigenous communities of Northwest Ontario and Anishnawbe Health Toronto, I found the article to be insightful. It reaffirmed commonly known facts about the issues facing Indigenous peoples in health care, such as privacy concerns, racism, discrimination and lack of culturally safe care.^2^ Although organizations such as the Indigenous Physicians Association of Canada have developed needed core competency for health care professionals^3^ and the Provincial Health Services Authority of British Columbia has developed courses to provide culturally safe care, the pace of change remains slow, and we continue to read stories about racism and discrimination against Indigenous people to this day. 

What is the root cause of these issues? In the late 1980s, when I taught Indigenous health to health sciences students at the University of Toronto, I asked my class to describe Indigenous people and one other racial group, such as Italians or Japanese in Canada. I was dismayed to find that nearly 90% of the adjectives cited for Indigenous people were stereotypically negative, compared to only 10% for the other racial group. Most students had no encounter with Indigenous people and based their opinions on media encounters.^4^ I realized that the students harboured “unconscious bias,” and to remedy this, they needed Indigenous cultural safety courses provided by Indigenous teachers who had “lived experiences.” I also conducted an environment scan to assess the teaching of Indigenous health courses across health sciences programs in Ontario’s colleges and universities and found a lack of such courses in most of them due to a lack of Indigenous teachers.^5^ With the help of Indigenous professionals, we developed an Indigenous cultural safety course and trained Indigenous preceptors across Ontario to deliver such courses in the health sciences programs. We found positive changes in students’ attitudes towards Indigenous people.^6^

What is needed is the education of all Canadians, young and old, as well as new and naturalized Canadians, about Indigenous history and the impact of colonization, residential schools, the Sixties Scoop and our postcolonial policies such as the *Indian Act* on the health and well-being of Indigenous peoples. To start with, I recommend reading the *Honouring the Truth, Reconciling for the Future* by the Truth and Reconciliation Commission of Canada.^7^

Thank you for bringing this issue to light. 
